# Measurement properties of the upright motor control test for adults with stroke: a systematic review

**DOI:** 10.1186/s40945-016-0027-z

**Published:** 2016-11-08

**Authors:** Edward James R. Gorgon, Rolando T. Lazaro

**Affiliations:** 1 Department of Physical Therapy, College of Allied Medical Professions, University of the Philippines, Pedro Gil Street, Malate, Manila 1004 Philippines; 2 Department of Physical Therapy, Samuel Merritt University, 450 30th Street 3726, Oakland, CA 94609 USA

**Keywords:** Clinical utility, Clinimetrics, Outcome measure, Reliability, Responsiveness, Validity

## Abstract

**Background:**

The Upright Motor Control Test (UMCT) has been used in clinical practice and research to assess functional strength of the hemiparetic lower limb in adults with stroke. It is unclear if evidence is sufficient to warrant its use. The purpose of this systematic review was to synthesize available evidence on the measurement properties of the UMCT for stroke rehabilitation.

**Methods:**

Electronic databases that indexed biomedical literature were systematically searched from inception until October 2015 (week 4): Embase, PubMed, Web of Science, CINAHL, PEDro, Cochrane Library, Scopus, ScienceDirect, SPORTDiscus, LILACS, DOAJ, and Google Scholar. All studies that had used the UMCT in the time period covered underwent hand searching for any additional study. Observational studies involving adults with stroke that explored any measurement property of the UMCT were included. The COnsensus-based Standards for the selection of health Measurement INstruments was used to assess the methodological quality of included studies. The CanChild Outcome Measures Rating Form was used for extracting data on measurement properties and clinical utility.

**Results:**

The search yielded three methodologic studies that addressed criterion-related validity and contruct validity. Two studies of fair methodological quality demonstrated moderate-level evidence that *Knee Extension* and *Knee Flexion* subtest scores were predictive of community-level and household-level ambulation. One study of fair methodological quality provided limited-level evidence for the correlation of *Knee Extension* subtest scores with a laboratory measure of ground reaction forces. No published studies formally assessed reliability, responsiveness, or clinical utility. Limited information on responsiveness and clinical utility dimensions could be inferred from the included studies.

**Conclusions:**

The UMCT is a practical assessment tool for voluntary control or functional strength of the hemiparetic lower limb in standing in adults with stroke. Although different levels of evidence suggest that the *Knee Extension* and *Knee Flexion* subtests may possess criterion and construct validity, the lack of published literature examining content validity, reliability, and responsiveness raises questions regarding the use of the UMCT in routine clinical practice. These key findings highlight the need to further investigate the UMCT’s measurement properties toward enhancing its standardization.

## Background

Significant impairment in lower limb strength is common after a stroke [1]. Impaired lower limb muscle strength is prominent in people who enter inpatient rehabilitation [2] and may persist in all muscle groups years after the stroke [3]. Literature supports the relationship between muscle weakness and post-stroke functional disability, especially in performing critical mobility tasks such as getting out of a chair, standing, walking, and negotiating stairs [1, 4–14]. Accurate evaluation of lower limb muscle weakness is therefore an essential component of effective stroke rehabilitation.

Dynamometry and manual muscle testing (MMT) are common measures of muscle strength. While isometric and isokinetic dynamometry has been demonstrated as an objective method of quantifying isometric and isokinetic strength in adults with stroke [15, 16], it requires special instrumentation which may not be feasible in settings where financial resources are limited. MMT was not designed for and cannot be used in persons with central nervous system lesions presenting with muscle tone alterations, abnormal reflex activity, abnormalities in amplitude, timing and scaling of synergistic muscle activity, and abnormal limb movement patterns [17–19]. There is therefore a need to identify a valid and clinically useful method of testing the strength of lower limb muscle groups that will not be limited by the presence of impaired muscle tone or inability to isolate joint movements.

The Upright Motor Control Test (UMCT) [20] or Upright Control (UC) Test [21–23] was originally developed as a clinician-administered clinical test of voluntary control of the affected lower limb in standing toward predicting functional walking ability in adults with stroke [C. Toman, unpublished thesis]. It is quick and simple to administer, requires no instrumentation and minimal physical space, and is therefore suitable for any clinical setting [24]. Clinically, it has been used to identify the presence of lower limb dyscontrol or muscle weakness in stroke [21, 23, 25, 26] and other neurological conditions [22, 24, 27]; and/or to measure stroke rehabilitation outcomes in longitudinal studies [19, 28], including clinical trials [29–31]. Since it is practical to administer and can be used on patients with muscle tone abnormalities and impairments in selective movement control from central nervous system lesions [20], it addresses the limitations related to dynamometry and MMT.

**Fig. 1:**
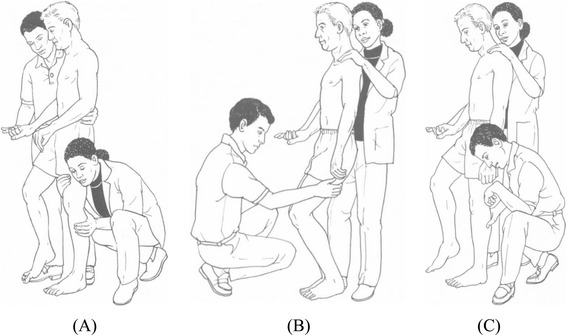
Upright Motor Control Test extension subtests. The UMCT comprises six extension and flexion subtests intended to reflect the limb loading and unloading demands of upright functional activities such as walking. Figure illustrates the extension subtests: hip extension (**a**), knee extension (**b**), and ankle plantarflexion (**c**). Reprinted with permission: Daniels and Worthingham’s Muscle Testing: Techniques of Manual Examination, 8th edition, Hislop HJ, Montgomery J, Upright Motor Control, pages 343–350, Copyright Elsevier (2007) [20]

The UMCT provides information on the ability to bear weight on and unload the affected lower limb in standing [19, 20]. The test can also assess both muscle force and muscle activation [19]. Weight bearing or extension control is assessed at the hip, knee, and ankle in the single-limb stance position (Fig. 1) [20], therefore simulating the limb loading requirements during stance phase of gait [C. Toman, unpublished thesis]. Unloading or flexion control is also assessed at the hip, knee, and ankle, while the contralateral lower limb is in single-limb stance [20]. The UMCT subtests, as well as the specific, required movements of the patient to complete the subtests, are detailed in Table 1.

**Table 1:** Upright motor control test components, subtest tasks, and scoring criteria

Test component	3 = Strong	2 = Moderate	1 = Weak
Extension Control Test			
*Hip Extension* Patient’s task: Stand on the weaker leg only and keep the body as straight as possible.	Maintains trunk erect on hip in single-leg stance	Unable to maintain trunk erect, but able to stop forward trunk momentum OR trunk wobbles back and forth OR trunk hyperextends on hip	Unable to control trunk flexion on hip in single-leg stance
*Knee Extension* Patient task: Stand on both legs with the knees bent, lift the stronger leg while the knee of the weaker leg remains bent, then straighten the knee of the weightbearing leg.	Supports body weight on flexed knee and straightens knee to end of range on command	Supports body weight on flexed knee (no further collapse into flexion)	Unable to maintain body weight on flexed knee (knee collapses in flexion)
*Ankle Extension* (*Plantarflexion*) Patient task: Stand on both legs with the knees straight, lift the stronger leg while the tibia of the weaker leg remains vertical, then raise the heel of the weaker leg as high as possible.	Maintains knee at neutral and lifts heel off floor on command	Can control knee at 0° and ankle at 90° with tibia vertical	Knee flexed, ankle dorsiflexed so that tibia displaces forward in single-leg stance
Flexion Control Test			
*Hip Flexion* Patient task: Bring the knee up toward the chest, as high and as fast as possible, while standing straight.	Actively flexes >60°, 3 rep within 10 s	Acively flexes 30-60°, 3 rep within 10 s	No motion OR actively flexes <30°, 3 rep taking >10 s
*Knee Flexion* Patient task: Bring the knee up toward the chest three times, as high and as fast possible, while standing straight.	Actively flexes >60°, 3 rep within 10 s	Actively flexes 30–60°, 3 rep within 10 s	No motion OR actively flexes <30°, 3 rep taking >10 s
*Ankle Flexion* (*Dorsiflexion*) Patient task: Bring the knee and foot up toward the chest as high and as fast as possible, while standing straight.	Actively dorsiflexes ≥90°, 3 rep within 10 s	Not used	No motion OR actively dorsiflexes <90°, 3 rep taking >10 s

Adapted from Hislop & Montgomery [20]

Generally, three *Extension Control* (*Hip Extension*, *Knee Extension*, and *Ankle Extension*) and two *Flexion Control* (*Hip Flexion* and *Knee Flexion*) subtests are rated on a three-point ordinal scale with the following muscle grade categories: *Strong*, *Moderate*, and *Weak* [20] (Table 1). The *Ankle Flexion* (*Dorsiflexion*) subtest comprises only two muscle grade categories: *Strong* and *Weak*. Subscale scores (*Strong* = 3, *Moderate* = 2, and *Weak* = 1) may be interpreted individually, as a combined extension or flexion score, or total (combined extension and flexion) score [C. Toman, unpublished thesis]. An additional category, *Excessive*, is applied for the knee extension and ankle extension (plantaflexion) subtests in cases of severe muscle tone impairment that preclude placing the test knee in flexion or the test ankle in plantigrade position [20]. To administer the UMCT, the clinician typically stands facing the patient, demonstrates each subtest to the patient to promote understanding, provides one or two practice trials, then observes and rates the patient’s performance based on the scoring criteria. Apart from being highly practical to administer, the UMCT also has a simple and well-defined scoring system.

Despite its advantages over conventional tests of muscle strength, little is known about the measurement properties of the UMCT for the population of patients with stroke. For a test to be acceptable for widespread use in both clinical practice and research, it must exhibit appropriate measurement or clinimetric properties such as reliability, validity, and responsiveness [32, 33]. This issue underscores the need to ascertain the usefulness of the UMCT in evaluating motor impairment and monitoring change over time to assess the impact of interventions. Therefore, the main purpose of this systematic review was to synthesize the available published literature on the measurement properties of the UMCT when used in adults with stroke. The review findings were projected to help in identifying research gaps that may warrant additional work to further develop and standardize the UMCT.

## Method

### Search strategy

Multiple electronic databases containing peer-reviewed literature were systematically searched from inception until October 2015 (week 4): Embase, PubMed, Web of Science, CINAHL, PEDro, Cochrane Library, Scopus, ScienceDirect, SPORTDiscus, LILACS, DOAJ, and Google Scholar. Keyword searching used the exact terms “upright motor control” and “upright control test”, which were the specific terms used to refer to the UMCT. All articles that described use of the UMCT, including any related literature review, underwent hand searching to locate additional studies. No restrictions were placed on publication language.

### Study selection

Two independent researchers (EJRG) and a trained research assistant (AL) implemented the search strategy, including full text examination of relevant studies. All titles and abstracts were screened, and all potentially relevant articles underwent full text examination. Peer-reviewed or published research articles reporting on measurement properties of the UMCT for adults (aged higher than 18 years) with stroke were included. Relevant studies were observational-methodologic in nature [34]. Exclusion criteria were: (1) study did not have an available full report or was available only as an abstract, such as publications in conference proceedings; (2) sample was a mix of neurological conditions with no separate clinimetric estimates reported for participants with stroke. To settle any disagreement, it was pre-planned that the independent researchers would re-examine the full-text article before arriving at a consensus.

### Quality assessment

The first author (EJRG) appraised data from the included studies using the COnsensus-based Standards for the selection of health Measurement INstruments (COSMIN) [35]. The COSMIN checklist comprises standardized criteria for evaluating the quality of methodological studies included in systematic reviews. Measurement properties that can be assessed on the COSMIN include: reliability, which is subdivided into internal consistency, reliability, and measurement error; validity, which comprises content validity (includes face validity), construct validity (covers structural validity, hypothesis testing, and cross-cultural validity), and criterion validity; and responsiveness. Each measurement property is examined using a number of quality criteria, with criteria pertaining to sample size and missing values being common across all the properties. Each item is rated on a four-point ordinal scale as “excellent”, “good”, “fair”, or “poor”. The COSMIN operates on the principle of “worst score counts”, i.e. the overall methodological quality score is determined by identifying the lowest or worst score among the items on the checklist [36]. The second author (RTL) independently verified the quality assessment. It was pre-planned that, should any disagreement arise, both authors would re-examine the article full text to arrive at a consensus.

### Data extraction

The first author (EJRG) performed data extraction using the CanChild Outcome Measures Rating Form [37]. This measure contains fields for extracting data on the measurement instrument’s focus based on the International Classification of Functioning, Disability and Health (ICF) [38]; scale construction; clinical utility; and standardization related to reliability, validity, and responsiveness. Additional information extracted from the selected studies include authors and publication year; test components assessed; purpose for applying the test; and sample characteristics such as sample size, age, gender, side of stroke, type of stroke based on etiology and chronicity, and functional status or severity of motor impairment. To enhance the accuracy of data extraction, explicit definitions of the measurement properties based on the COSMIN [39] were adopted (Table 2). The second author (RTL) independently verified the data extraction. As with quality assessment, for any disagreement that would arise, it was pre-planned that both authors would re-examine the full-text and arrive at a consensus.

**Table 2:** Definitions of and standards used to interpret measurement properties

Property	Definition [39]	Standard for interpretation [40]
Reliability	The extent to which scores for patients who have not changed are the same for repeated measurement under several conditions: e.g. over time (test-retest); by different persons on the same occasion (inter-rater) or by the same persons (i.e. raters or responders) on different occasions (intra-rater)	+ = ICC or weighted Kappa ≥0.70 ? = Doubtful design or method (e.g. time interval not mentioned) - = ICC or weighted Kappa <0.70, despite adequate design and method
Validity: Construct validity (hypotheses testing)	The degree to which the scores of a measurement are consistent with hypotheses (for instance with regard to internal relationships, relationships to scores of other instruments or differences between relevant groups) based on the assumption that the measurement validity measures the construct to be measured	+ = Correlation with an instrument measuring the same construct ≥0.50 OR at least 75 % of the results are in accordance with these hypotheses AND correlation with related constructs is higher than with unrelated constructs ? = Solely correlations determined with unrelated constructs - = Correlation with an instrument measuring the same construct <0.50 OR <75 % of the results are in accordance with the hypotheses OR correlation with related constructs is lower than with unrelated constructs
Validity: Criterion validity	The degree to which the scores of a measurement are an adequate reflection of a “gold standard”	+ = Convincing arguments that gold standard is “gold” AND correlation with gold standard ≥0.70 ? No convincing arguments that gold standard is “gold” OR doubtful design or method - = Correlation with gold standard <0.70, despite adequate design and method
Responsiveness	The ability of a measurement to detect change over time in the construct to be measured	+ = Correlation with an instrument measuring the same construct ≥0.50 OR at least 75 % of the results are in accordance with these hypotheses OR AUC ≥0.70 AND correlation with related constructs is higher than with unrelated constructs ? = Solely correlations determined with unrelated constructs - = Correlation with an instrument measuring the same construct <0.50 OR <75 % of the results are in accordance with the hypotheses OR AUC <0.70 OR correlation with related constructs is lower than with unrelated constructs

*ICC* intraclass correlation coefficient, *AUC* area under the curve, + positive rating, ? indeterminate rating, − negative rating

Doubtful design or method = lacks clear description of study design or methods; used sample size smaller than 50 participants; or any important methodological flaw in study design or implementation

### Data analysis and synthesis

The authors performed a best evidence synthesis based on the COSMIN guidelines. For each measurement property, the possible overall rating was “positive”, “indeterminate”, or “negative” (Table 2) [40]. This overall rating was accompanied by an assessment of the level of evidence based on the work of the Cochrane Back Review Group: “strong”, “moderate”, “limited”, “conflicting”, or “unknown” (Table 3) [41]. The levels of evidence are determined based on the number of studies that have investigated the measurement property, methodological quality of such studies, and consistency of the results of such studies.

**Table 3:** Levels of evidence for quality of measurement properties proposed by Cochrane Back Review Group

Level	Rating	Criterion
Strong	+++ or ---	Consistent findings in multiple studies of good methodological quality OR in one study of excellent methodological quality
Moderate	++ or --	Consistent findings in multiple studies of fair methodological quality OR in one study of good methodological quality
Limited	+ or -	One study of fair methodological quality
Conflicting	+/−	Conflicting findings
Unknown	?	Only studies of poor methodological quality

+ positive rating, ? indeterminate rating, − negative rating

Adapted from van Tulder et al. [41]

## Results

### Search results

**Fig. 2:**
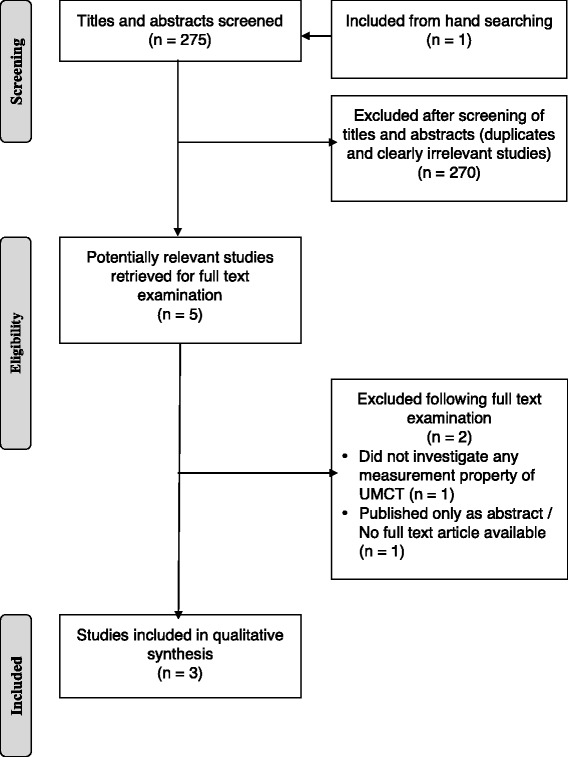
Flow of studies in the literature search

The search yielded a total of 275 citations (Fig. 2). Since use of single keywords generated manageable search yields, use of keyword combinations was unnecessary. Initial screening was done to remove duplicates. Next, abstracts were examined to exclude studies that did not investigate any measurement property of the UMCT. Following this step, five titles remained for full-text review. Two articles were further excluded because one study used the UMCT as a descriptive measure only and did not explore any aspect of instrument development [21], and the other study investigated interrater reliability but was published only as a conference abstract with no full report available [42]. Three studies examined the UMCT’s measurement properties [43–45] and were included in the qualitative synthesis. The researchers were in full agreement on the selection of the final studies to be included.

### Included studies

Table 4 shows the characteristics of the participants in the included studies. Sample sizes ranged from 33–147 adults with stroke, while mean ages ranged from 53.9–58.73 years. Samples had a mix of genders, sides of hemiplegia, and types of stroke based on etiology (ischemic and hemorrhagic). Two studies included both subacute and chronic stroke populations [43, 44], while one study included only patients with subacute stroke (30–180 days post-onset) [45].

**Table 4:** Characteristics and findings of included studies on upright motor control test measurement properties

Authors	Participants	Test component assessed	Purpose of test application	Validity	Clinical utility
Joa et al. [43]	124 adults with subacute and chronic strokes (56 F; 73 ischemic stroke; 68 left hemiplegia) Age = 53.9 (SD 15.4) yr 66 community walkers; 58 household walkers	*Knee Extension*, *Knee Flexion*	To test voluntary control of hemiparetic lower limb	Criterion validity (diagnostic accuracy) assessed Subtests predictive of community-level ambulation versus home-level ambulation • Score of 3 (*Strong* score) for either *Knee Extension* or *Knee Flexion* predicted community ambulation • Score of 1 or 2 (*Weak* or *Moderate* score) for both *Knee Extension* and *Knee Flexion* predicted household ambulation Sensitivity, specificity, and AUC for identifying restriction in community ambulation • UMCT alone: 98 %, 67 %, and 0.829 • UMCT combined with Korean BBS: 81 %, 93 %, and 0.875 • UMCT combined with gait velocity: 89 %, 91 %, and 0.904 • UMCT combined with Korean BBS and gait velocity: 80 %, 94 %, and 0.876	Does not require equipment Takes approximately 1 min to complete (each test)
Perry et al. [44]	147 adults with subacute and chronic strokes (79 F; different etiologies; 79 left hemiplegia) Age = 55.5 (SD 12.2) yr 78 community walkers; 69 household walkers	All 6 components of *Extension Control Test* and *Flexion Control Test*	To test functional muscle strength of hemiparetic lower limb	Criterion validity (predictive validity) assessed Composite scores not significantly different across 6 functional walking categories Scores on *Knee Extension* and *Knee Flexion* predictive of community-level ambulation versus home-level ambulation • 78 % accuracy, 78 % agreement • Score of 3 (*Strong* score) for either *Knee Extension* or *Knee Flexion* predicted community ambulation • Score of 1 or 2 (*Weak* or *Moderate* score) for both *Knee Extension* and *Knee Flexion* predicted household ambulation • *Knee Extension* subtest combined with gait velocity: 87 % agreement with expert clinicians in differentiating between community ambulators and household ambulators • Score of 3 (*Strong* score) for *Knee Extension* with gait velocity of at least 16 m/min predicted community ambulation • Scores of 1 and 2 (*Moderate* and *Weak* scores) for *Knee Extension* with gait velocities lower than 32 m/min and 24 m/min respectively predicted household ambulation	No specific information provided
Mercer et al. [45]	33 adults with subacute stroke (15 F; 23 left hemiplegia); 25 completing all 6 testing sessions Age = 58.73 (SD 17.27) yr Baseline FMA lower limb motor scale score = 17.82 (SD 6.22)	*Knee Extension*	To test voluntary control of hemiparetic lower limb	Construct validity (convergent validity) assessed Positive correlations between *Knee Extension* scores and paretic-limb peak vertical GRF (pseudo R2) • 0.34, during stepping with non-paretic limb • 0.22, during diagonal reach task • 0.21 = during sit-to-stand task	Easily administered in a variety of clinical settings

*AUC* area under the received operator characteristic curve, *BBS* Berg Balance Scale, *FMA* Fugl-Meyer Assessment, *GRF* ground reaction force, *ST* Step Test, *UMCT* Upright Motor Control Test

### Quality assessment

Given the COSMIN’s use of the “worst score counts” principle, all studies had an overall rating of “fair” (Table 5). The *Criterion validity* subscale was used to assess the two studies on predictive validity [43, 44], while the *Hypothesis testing* (*Construct validity*) subscale was used on the study on convergent validity [45]. Evidence for criterion validity was positive but the two supporting studies shared similar limitations. Both had insufficient description of missing data and information to justify the choice of the gold standard, therefore resulting in a “fair” quality rating [43, 44]. Evidence for construct validity, though positive, was limited by the supporting study’s lack of a clear a priori hypothesis, and insufficient description of the comparator instrument and its measurement properties [45]. Therefore, a “fair” quality rating was given.

**Table 5:** Methodological quality of included studies and levels of evidence for quality of measurement property

Study	Hypothesis testing (Construct validity)	Criterion validity	Reliability	Responsiveness
Joa et al. [43]		Fair / ++		
Perry et al. [44]		Fair / ++		
Mercer et al. [45]	Fair / -			
*Level of evidence*	*Limited*	*Moderate*		

Level of evidence [41]: Strong = consistent findings in multiple studies of good methodological quality OR in one study of excellent; Moderate = consistent findings in multiple studies of fair methodological quality OR in one study of good methodological quality; Limited = one study of fair methodological quality methodological quality; Conflicting = Conflicting findings; Unknown = Only studies of poor methodological quality

+ positive rating, ? indeterminate rating, − negative rating

### Data extraction and synthesis

Table 4 summarizes the results of the included studies. One study covered all six *Extension Control* and *Flexion Control* subtests [44]; one delimited investigation to the *Knee Extension* and *Knee Flexion* subtests only [43]; and one focused on the *Knee Extension* subtest only [45]. The studies used the UMCT as a test of voluntary control [43, 45] or functional strength [44] of the affected lower limb, covering the *Body Functions* dimension of the ICF framework (*Power of Muscles of One Side of the Body*, ICF code b7302) [38]. The three studies addressed dimensions of validity, but none assessed reliability. None of the published studies addressed content validity, particularly aspects of scale construction such as selection of test items for inclusion and weighting of items in scoring. One study described some observations regarding responsiveness [45], while two studies provided information related to some clinical utility dimensions [43, 45].

Two studies on criterion (predictive) validity reported similar findings that the *Knee Extension* and *Knee Flexion* subtests differentiated between community and household ambulators [43, 44]. Both studies demonstrated that a *Strong* score on either subtests could be used to identify community ambulators while a *Moderate* or *Weak* score on both subtests could be used to identify household ambulators. Further, both studies demonstrated the predictive ability of the knee subtests when combined with other clinical measures such as gait velocity [43, 44] and the Korean version of the Berg Balance Scale (BBS) [43]. A *Strong* score on the *Knee Extension* subtest combined with a minimum gait velocity of 16 m/min characterized community ambulators in one study [44], while the knee subtests, applied alone or with either or both gait velocity or the BBS, yielded areas under the receiver operating characteristic curve (AUC) of 0.829–0.904 in the other study [43]. One study on construct (convergent) validity found that *Knee Extension* scores positively correlated with peak vertical ground reaction force measurements during limb loading tasks such as sit-to-stand, diagonal reaching, and stepping with the non-paretic lower limb (psuedo *R* ^2^ = 0.21–0.34) [45]. The levels of evidence represented by the included studies were “moderate” for criterion validity and “limited” for construct validity (Table 5).

Although none of the studies formally assessed responsiveness and clinical utility, data were available related to these properties. One study reported that improving *Knee Extension* scores were related to increasing Step Test scores over the first 6 months post-stroke [45]. From 73 % (24/32) at 1 month post-stroke, the proportion of participants with a score of 1 (*Weak* score) decreased to 31 % (9/29) at 6 months post-stroke. From 12 % (4/32) at 1 month post-stroke, the proportion of participants with a score of 3 (*Strong* score) rose to 55 % (16/29) at 6 months post-stroke. In the same study, floor and ceiling effects were also reported, with 27 % (9/33) of participants not improving from a *Weak* score even after 6 months and 36 % (12/33) achieving the highest score (*Strong* score) before the last testing session. Two studies mentioned data addressing some clinical utility dimensions: ease of administration [45]; no special equipment required [43]; and short administration time [43].

## Discussion

This review synthesized the evidence on the measurement properties of the UMCT from three studies on validity [43–45] located through a comprehensive literature search. Best evidence synthesis indicates that there is moderate level of evidence to support criterion validity of the *Knee Extension* and *Knee Flexion* subtests and limited level of evidence for construct validity of the *Knee Extension* subtest. The results of this synthesis, together with the lack of literature formally assessing content validity, reliability, and responsiveness, provide an important basis for evaluating the current usefulness of the UMCT as well as identifying important knowledge gaps for further research.

Evidence for different dimensions of validity synthesized in this systematic review is consistent with the original work on the UMCT. Validity of the UMCT was first examined in 1983 in an unpublished master’s thesis [C. Toman, personal communication]. In that study, relationships between the UMCT score (then called the UC Test) and gait variables were evaluated in 20 adults with subacute or chronic stroke. Total UC scores from the 6 subtests correlated significantly with important gait parameters such as gait velocity, stride length, and single-limb support time in the hemiparetic lower limb. This significant correlation with gait parameters is in keeping with moderate-level evidence from this review that the UMCT, specifically the *Knee Extension* and *Knee Flexion* subtests, can be used to predict walking ability in adults with subacute or chronic stroke [43, 44].

In one study [43], when the *Knee Extension* and *Knee Flexion* subtests were used either alone or in combination with gait velocity, the BBS, or both, the range of AUC values reported suggests at least moderate accuracy [46] in separating individuals with walking restrictions (household ambulators) from those without walking restrictions (community ambulators). Therefore, the available evidence is in agreement with the assertion of Perry et al. that the knee subtests may represent a valid method of assessing voluntary total limb control in standing [44]. Still, however, the exact process employed in the development of the UMCT’s subtests and scales, and interpretation of cumulative scores remain unclear and therefore warrants examination for content validity.

The absence of published reports on the UMCT’s reliability has important negative ramifications on the accuracy of its validity estimates. Reliability estimates for the UMCT when used on patients with stroke-related hemiplegia have been mentioned in earlier work involving clinicians [J. Montgomery, unpublished data] or students [42], however such work was never published in a peer-reviewed journal. Thus, sufficient study appraisal and data extraction could not be carried out. Without interrater reliability estimates, it is uncertain if measurements would be stable across different raters. In practice, clinicians who might use the UMCT would likely possess varied clinical practice experience and the impact of such differences in practice experience would be important to know. Also, without test-retest reliability estimates, it is unclear if measurements would be stable in longitudinal assessment in the absence of real change from either spontaneous recovery or the effects of intervention. This key research gap highlights the need to examine the interrater and test-retest reliability of the UMCT in adults with stroke.

The lack of studies that formally assess responsiveness can have a negative impact on the UMCT’s value in longitudinal assessment. Limited data from one study suggests that, although scores on the *Knee Extension* subtest may change over time, possible floor and ceiling effects can be observed [45]. This finding may be related to the few scale levels available for scoring which might diminish the instrument’s ability to detect small changes in performance. One clinical trial [31] has demonstrated that scores on the UMCT can significantly change over time in adults with stroke exposed to an active treatment compared to those exposed to placebo. However, studies on effectiveness of interventions are inappropriate when demonstrating responsiveness [39]. Studies that can demonstrate that scores on the test of interest (i.e. UMCT) change correspondingly with scores on a test that is considered a gold standard would be more appropriate for addressing the knowledge gap [39]. Such studies are important in light of research literature in which the UMCT has been used in longitudinal assessment.

Although no study has formally assessed clinical utility, data inferred from existing literature indicate that features of UMCT administration are consistent with known features of highly practical tests in clinical practice [37]. This finding highlights an important advantage of the UMCT over measures that are well established but require expensive instrumentation that preclude use in many clinical settings such as dynamometry. Additionally, the available studies on the UMCT’s measurement properties did not require the exclusion of participants who were incapable of selective limb control. This feature emphasizes a key advantage of the UMCT over extensively used tools such as dynamometry and MMT. Thus, current evidence on measurement properties and practicality of test administration provide an argument for the potential of the UMCT to be further developed and standardized.

This systematic review has limitations. At the review level, few published studies have investigated the measurement properties of the UMCT to date. Thus, firm conclusions regarding most of the UMCT’s properties, especially content validity, reliability, and responsiveness, cannot be made at this point. At the study level, the available studies have reported on the properties of the *Knee Extension* [43–45] and *Knee Flexion* subtests [43, 44] only, and the methodological quality of the studies was fair [43–45]. Although the *Knee Extension* subtest procedure and scoring was applied consistently across the three studies [43–45], the *Moderate* score of the *Knee Flexion* subtest was omitted in one of the two studies that examined it [44]. Therefore, further enhancement of the methodological quality of studies and clarification of the application of the scoring method are warranted.

## Conclusion

Findings of this systematic review indicate that in adults with subacute and chronic types of stroke, moderate evidence from two studies supports that the *Knee Extension* and *Knee Flexion* subtests of the UMCT can potentially identify adults with restrictions in functional walking. Further, in adults with subacute stroke, limited evidence from one study suggests that the *Knee Extension* subtest positively correlates with weight bearing. Since no instrumentation is needed, administration is easy, and time and space requirements are minimal, it is feasible for clinicians to routinely apply the UMCT in practice. However, its use in clinical practice and research is limited by the absence of published data on content validity, reliability, and responsiveness. Further research should assess these important measurement properties of the UMCT to support its integration in stroke rehabilitation.

## Abbreviations

AUC: Areas under receiver operating characteristic curve
BBS: Berg balance scale
COSMIN: COnsensus-based Standards for the selection of health Measurement INstruments
ICF: International classification of functioning, disability and health
MMT: Manual muscle testing
UC: Upright control
UMCT: Upright motor control test
